# Effects of Enzymatic Modification and Cross-Linking with Sodium Phytate on the Structure and Physicochemical Properties of *Cyperus esculentus* Starch

**DOI:** 10.3390/foods11172583

**Published:** 2022-08-25

**Authors:** Jun Wang, Dejian Zhang, Jiechuan Xiao, Xiaotong Wu

**Affiliations:** School of Life Sciences, Inner Mongolia University, Hohhot 010031, China

**Keywords:** *Cyperus esculentus*, enzyme modification, sodium phytate, cross-linking

## Abstract

In this study, *C. esculentus* porous starch (PS) and *C. esculentus* cross-linked porous starch (CPS) were prepared by enzymatic modification and sodium phytate cross-linking, and their physicochemical and structural properties were determined. The results showed that the adsorption and emulsification capacities of PS were 1.3606 g/g and 22.6 mL/g, respectively, which were significantly higher than 0.5419 g/g and 4.2 mL/g of *C. esculentus* starch (NS). The retrogradation curves of starch paste showed that the stability of PS was inferior to that of NS. In addition, the results of texture analysis showed that the gel strength of PS was also significantly reduced relative to NS. The PS exhibited a rough surface with pores and low molecular order and crystallinity according to scanning electron microscope (SEM), fourier infrared spectroscopy (FTIR), and X ray diffractometer (XRD) analyses. As compared to PS, CPS still presented a high adsorption capacity of 1.2744 g/g and the steadiness of starch paste was significantly better. XPS demonstrated the occurrence of the cross-linking reaction. Our results show that enzyme modification and dual modification by combining enzymatic treatment with sodium phytate cross-linking can impart different structures and functions to starch, creating reference material for the application of modified starch from *C. esculentus*.

## 1. Introduction

*Cyperus esculentus* is an annual herb in the sedge family, which is also known as tiger nut and iron water chestnut, is widely cultivated in tropical and subtropical regions because of its high resistance to adversity and low soil requirements. Many studies have demonstrated that *C. esculentus* is rich in nutritional value and is a crop with a high yield and comprehensive use-value of its oil and grain; it has thus gained the reputation of an “underground walnut”. It is one of the most promising healthcare-related genera in the family Salviaceae, which is rich in bioactive substances and has been studied for pharmacological benefits [1,2,3]. In recent years, it has attracted more and more attention in the scientific community [4].

Starch is a low-cost, readily available, biodegradable, and non-toxic natural polymer material. However, its inherent properties, such as high retrogradation tendency, water insolubility, and poor thermal stability limit its application in food, medicine, and chemical industries [5]. The starch content of *C. esculentus* can be 20% or more. It has been shown that the surface of starch granules is smooth. They are mostly spherical or oval with particle diameters ranging from 3.41 to 12.12 μm and an average particle size of 8.60 μm. They have a typical A-type crystal structure with high thermal stability. In addition, as compared to potato starch, *C. esculentus* starch paste has lower viscosity and transparency and better freeze–thaw stability [6,7].

Porous starch (PS), as a type of modified starch, has been receiving increasing attention because of its valuable functions and potential applications. At present, the methods of preparing PS include physical, chemical, enzymatic, and synergistic methods. The enzymatic method is more acceptable because of its mild reaction conditions, high efficiency, and specificity [8]. The properties of PS are closely related to the natural starch granule source and particle size [9]. The porous modification of starch from corn, potato, and wheat has been reported so far. However, PS has some inherent defects, such as low shear resistance and deteriorated thermal stability. Therefore, in this experiment, we attempted to modify the PS of *C. esculentus* by enzyme modification and to investigate the changes in these properties.

Being an important means of starch modification, cross-linking changes the overall structure and surface chemistry of starch by introducing chemical bonds at random locations in the starch granules. It can stabilize the starch granule structure and can enhance acid, heat, and shear resistance. Thus, it is often used in conjunction with the porous modification of starch to improve its properties [10,11]. Triclosan, acetic acid, sodium trimetaphosphate, and sodium tripolyphosphate are frequently used as cross-linking agents in the food industry [12]. Phytic acid is a natural antioxidant consisting of an inositol ring and six hydrophilic phosphate groups. It has been widely used in food, pharmaceutical, and daily chemical applications [13,14]. A study by Sun et al. using sodium phytate as a cross-linking modifier confirmed that sodium phytate cross-linking enhanced the connection between wheat starch particles, made the starch arrangement more ordered, significantly increased the viscosity coefficient, and demonstrated excellent freeze-thaw stability [15]. However, to date, no PS modification with sodium phytate has been reported in the literature. In this experiment, the effect of sodium phytate cross-linking on the properties of PS was investigated based on the successful porous modification of *C. esculentus* starch.

## 2. Materials and Methods

### 2.1. Materials

The purity of *C. esculentus* starch must be > 90%, which was extracted from *C. esculentus* meal (Jilin Hao Yi Cai Oil Co., Ltd., Jilin, China) by the alkaline extraction method [3]. The α-amylase, amyloglucosidase from *Aspergillus niger*, and sodium phytate were purchased from Sinopharm Chemical Reagent Co., Ltd., Beijing, China. Potato amylose was purchased from Shanghai Yuanye Biotechnology Co., Ltd., Shanghai, China. All reagents were analytically pure, unless indicated otherwise.

### 2.2. Preparation of Samples

#### 2.2.1. Synthesis of *C. esculentus* Porous Starch (PS)

A buffer solution containing 0.1 M citric acid and 0.2 M disodium hydrogen phosphate at pH 5 was prepared in advance and preheated. It was mixed with *C. esculentus* starch in the ratio of 1:8, and the enzyme complex (3%, w:w, α-amylase: amyloglucosidase from *A. niger* = 1:3) was added. The reaction was then carried out on a magnetic stirrer (ZWCL-TS, Shanghai, China) for 10 h (50 °C, 160 rpm); it was then terminated by adding 0.1 M sodium hydroxide solution to adjust the pH of the starch suspension to 10 according to previous studies [11]. The starch suspension was centrifuged (4200 rpm for 10 min), and the precipitate was washed repeatedly with distilled water for neutralization. Finally, it was dried in a blast dryer at 50 °C.

#### 2.2.2. Synthesis of *C. esculentus* Cross-Linked Porous Starch (CPS)

Cross-linking of *C. esculentus* porous starch with sodium phytate was carried out by using the method of Sun et al., with slight modification [15]. Briefly, *C. esculentus* porous starch was used to prepare a 20% starch suspension in distilled water. The sodium phytate solution was then added slowly while stirring at 1% (w:w) as the cross-linking agent. The pH of the starch suspension was adjusted to 7, and the reaction temperature was kept at 50 °C. After 6 h of reaction, the contents were centrifuged at 4200 rpm for 10 min (5804R, Eppendorf Co., Ltd., Hamburg, Germany), and the precipitate was washed several times with distilled water until the supernatant did not produce a white turbid substance with 0.1 M silver nitrate solution, to ensure that free sodium phytate was not contained in CPS. Finally, the precipitate was dried in a blast drying oven at 50 °C to obtain the cross-linked porous starch (CPS) of *C. esculentus*.

### 2.3. Determination of Physical and Chemical Properties

#### 2.3.1. Adsorption Properties

The oil absorption capacity was evaluated by using the method described by Li et al. [12]. An accurately weighed sample of starch (*M*_0_) was placed in a 50 mL centrifuge tube (*M*_1_) of known mass. A certain amount of soybean oil was added and mixed well using a vortex mixer. The contents were allowed to stand for 30 min until the adsorption equilibrium was achieved. The contents were centrifuged at 4200 rpm for 20 min, and the oil layer was poured off until no more oil drops fell. The total mass of the centrifuge tube and solids (*M*_2_) was determined. The oil absorption of porous starch was calculated using Equation (1).

(1) $$A_{\left(g/g\right)}=\frac{M_{2}-M_{1}-M_{0}}{M_{0}}$$

#### 2.3.2. Emulsification Capacity

A gram of starch was configured into a starch suspension with a mass fraction of 1%. The contents were placed in a boiling water bath with stirring for 20 min to allow the starch to disperse. An equal volume of soybean oil was homogenized for 3 min and then dispensed into 10 mL centrifuge tubes. Centrifugation at 3000 rpm for 20 min caused visible stratification, and the amount of floating oil was measured. The volume of oil emulsified per gram of starch sample was calculated [16].

#### 2.3.3. Transparency

The starch suspension was prepared according to the material–liquid ratio of 1:100. It was heated and stirred in a boiling water bath for 30 min, while adding boiling water continuously to keep the volume of starch suspension constant. After cooling to room temperature, the light transmittance was measured at a wavelength of 650 nm, with distilled water as a reference [3].

#### 2.3.4. Starch Paste Stability

A starch suspension with a mass concentration of 1% was prepared. It was heated in a boiling water bath and stirred for 30 min to obtain a uniform paste, which was poured into a 50-mL graduated test tube to cool. After that, the suspension was stored in a refrigerator at 4 °C, and the change in supernatant volume was recorded within 24 h [13].

#### 2.3.5. Amylose Content

The determination of amylose content was done by the method of Castro et al. [17]. The samples were treated by the methanol reflux degreasing method. A standard was prepared with pure potato amylose and a calibration curve was drawn. Finally, the iodine colorimetric method was used to determine the amylose content.

#### 2.3.6. Paste Properties

The starch samples were ground in a mortar after moisture equilibration and then passed through a 100-mesh sieve. Twenty-five milliliters of 10% starch dispersion (*w*/*w*, dry basis) were prepared and transferred to an aluminum canister. The viscosity was measured using a rapid viscosity analyzer (RVA Super 4, Newport Scientific, Irvine, USA), whose procedure has been detailed by Sun et al. [15].

#### 2.3.7. Gel Properties

Next, 6% (w:w) starch suspension was heated in a water bath for 30 min with constant stirring. After cooling for 24 h (at 4 °C) to form a stable gel, the contents were taken out from the beaker (diameter = 40 mm) and were transferred into a cylinder with a bottom diameter of 40 mm and a height of 15 mm. The surface of starch was uniform and flat. The hardness, springiness, glueyness, and chewiness of the starch gels were determined using a texture analyzer (TMS-Touch, FTC Inc., Austin, TX, USA). The TPA test model was used to determine the starch gel properties using a P/75 disc probe with pre-, mid-, and post-measurement rates set to 0, 30, and 60 mm/min. The time interval between two compressions was set to 2S, the compression ratio was set to 20%, and the trigger force was set to 0.05 N [18].

### 2.4. Structural Characterization

#### 2.4.1. Scanning Electron Microscopy (SEM) Analysis

The morphology of starch particles was observed under SEM (S-4800, Hitachi Int., Tokyo, Japan). First, a small amount of starch sample was evenly spread on the conductive gel on the carrier table. Excess starch was then blown off with an ear wash ball, and the stage was put into the gold plating apparatus for a minute. Finally, the sample was put under the SEM for observation. A clear image was obtained at 2500× magnification.

#### 2.4.2. Differential Scanning Calorimetry (DSC)

A starch sample (3 mg) was placed in a stainless-steel dish, and 7 mg of distilled water was added to it. The dish was then sealed and equilibrated at room temperature for 24 h. The thermal properties of starch were analyzed by DSC (DSC3, Mettler Toledo Technology Co., Ltd, Zurich, Switzerland). DSC was performed as follows: temperature increase rate of 10 °C/min for a temperature range of 30–120 °C. Changes in the onset pasting temperature (To), peak temperature (Tp), conclusion temperature (Tc), and enthalpy (ΔH) were measured separately [19].

#### 2.4.3. X-ray Diffraction Analysis (XRD)

X-ray diffraction patterns of the three types of starch were obtained using the Bruker D8 Advance diffractometer (Bruker AXS Inc., Karlsruhe, Germany) with Cu-Ka as the characteristic ray. Data was acquired with a scan range of 5–40° (2θ) at a scan rate of 5°/min (40 kV and 40 mA) [20].

#### 2.4.4. Fourier Transform Infrared Spectroscopy (FT-IR) Analysis

The FT-IR spectra of the starch samples were obtained by an FT-IR spectrophotometer (IRAffinity-1S, Shimadzu, Kyoto, Japan). The starch sample was mixed with KBr in a certain ratio (1:100), and then poured into an agate mortar, ground to a powder, and transferred to a tablet press to be pressed into transparent flakes [15]. KBr-pelletized starch samples were analyzed in the range of 4000-400 cm^−1^ with a resolution of 4 cm^−1^ and 64 scans using a thin slice of KBr as a background.

#### 2.4.5. X-ray Photoelectron Spectroscopy (XPS)

Powdered starch samples were spread on double-sided tape on a sample table to analyze the structure and surface elemental composition of starch samples by in situ XPS (ESCALABXI+, Thermo Scientific Co., Waltham, MA, USA) at a passage energy of 23.5 eV and a resolution of 0.05 eV [21].

#### 2.4.6. Specific Surface Area and Pore Size (BET)

The starch samples were degassed under vacuum at 120 °C for 8 h to remove adsorbed gas molecules and residual solvent from the sample surface. A rapid specific surface area and pore size distribution tester (ASAP2020, McMuratic Instruments Co., Ltd, Shanghai, China) were used to determine the N_2_-sorption/desorption isotherms of starch samples. The specific surface area and pore size distribution of the starch samples were calculated by multi-point BET (Brunner–Emmet–Teller) and BJH (Barret–Joyner–Halenda) methods, respectively [19].

### 2.5. Statistical Analysis

All experiments were repeated at least three times. Results were expressed as mean ± standard deviation (SD) and analyzed using the software, Origin 2018 (Stat-Ease Inc., Minneapolis, MN, USA). Statistical analysis was performed using SPSS 22 (SPSS Inc., Chicago, IL, USA). One-way analysis of variance (ANOVA) and Duncan’s multiple range test was used to determine significant differences (*p* < 0.05).

## 3. Results and Discussion

### 3.1. Physicochemical Properties

#### 3.1.1. Changes in Basic Physical Properties

In the present study, some physical properties of *C. esculentus* starch and its modified starch were determined (Table 1). The oil absorption capacities of the NS, PS, and CPS were 0.5419, 1.3606, and 1.2744 g/g, respectively. After enzymatic modification, the oil absorption capacity of the starch exhibited a significant increase, which was due to the destruction of the original structure of starch and the appearance of pores upon enzymatic modification [11]. The increased number of pores offered more binding sites and increased the adsorption capacity of starch. Emulsification ability is an important property that determines the applicability of starch. As shown in Table 1, the emulsification capacity of PS was 22.6 mL/g, which was significantly higher than that of NS (4.2 mL/g). This is because PS has a large specific surface area, which can significantly enhance the adsorption capacity of oil. However, compared with PS, the emulsification ability of CPS was significantly reduced due to the introduction of hydrophilic groups.

The transparency of starch paste, which is usually expressed in terms of transmittance, directly affects the sensory acceptability of starchy foods and reflects the strength of the starch to bind with water. The transmittance of the three starch pastes is presented in Table 1, where both enzymatic and cross-linking modifications significantly improved the transparency of the starch paste. The reduced emulsification capacity, as well as the increased transparency, of the cross-linked starch were mainly due to the introduction of hydrophilic phosphate groups due to cross-linking, which increased the hydrophilic capacity of the starch [22].

#### 3.1.2. Starch Retrogradation Analysis

**Figure 1:**
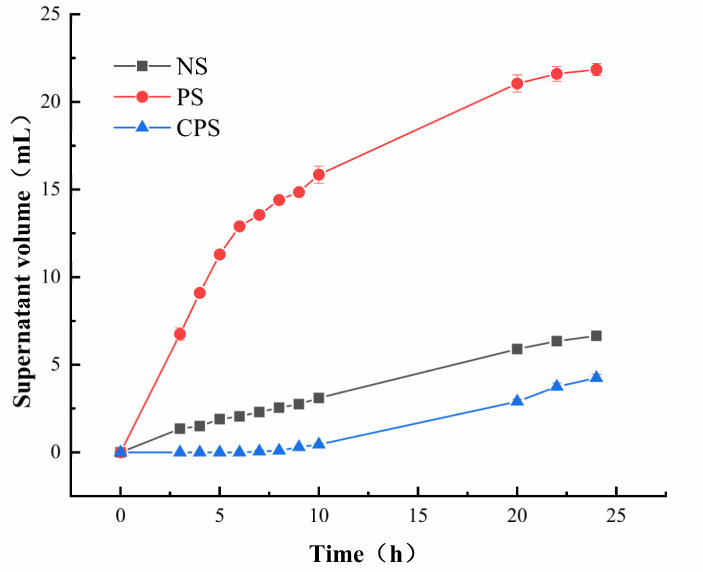
Retrogradation curves. NS: native *Cyperus esculentus* starch; PS: *Cyperus esculentus* porous starch; CPS: *Cyperus esculentus* cross-linked porous starch.

When gelatinized starch is placed at a low temperature, starch molecules rearrange and form insoluble precipitates through hydrogen bonding, in a process called retrogradation. The retrogradation stability of starch is usually measured by the percentage of liquid supernatant during the placement of starch paste. Since the volume of starch paste in all samples in this experiment is the same, it is directly expressed by the volume of liquid supernatant. The retrogradation curves of *C. esculentus* starch and its modified form are shown in Figure 1. The retrogradation stability of NS deteriorates after enzyme modification, while that of PS increased significantly after cross-linking. This is mainly due to enzymatic damage to the starch molecular structure, which allowed the starch to age readily. However, cross-linking introduced hydrophilic phosphoric acid groups. The introduction of phosphoric acid groups not only promoted the formation of a more stable three-dimensional network between the sub-molecular chains of starch, but also improved the hydration of starch molecules after pasting. The intermolecular interactions are beneficial for the starch paste system to maintain stability [22,23].

#### 3.1.3. Pasting Properties

The pasting viscosity parameters of *C. esculentus* starch and its modified form are summarized in Table 2. As compared to the peak viscosity (PV, 235.50 mPa.s), trough viscosity (TV, 231.00 mPa.s), and final viscosity (FV, 233.00 mPa.s) of the NS, these parameters decreased to 69.68, 72.00, and 88.60 mPa.s, respectively, for PS. This could be due to the enzymatic disruption of the starch granules to produce a greater number of short-branched straight chains, which could have weakened the inter-chain aggregation [18]. However, the viscosity of starch paste increased after cross-linking of PS, leading to a PV, TV, and FV of 121.00, 113.33, and 116.67 mPa.s, respectively. The increase is mainly attributed to an introduction of hydrophilic phosphate functional groups, resulting in more water absorption.

As shown in Table 2, the breakdown viscosity (BD) of PS was significantly lesser than that of NS and CPS. The lower BD of enzymatically modified starch could be attributed to the higher amylose content and longer linear chains in it. In addition, it is possible for amylose to form helical complexes with lipids. Furthermore, long linear chains can intertwine with one another. Both these alterations are likely to inhibit the swelling of starch to maintain the integrity of its granules during heating [24].

According to the study by Dura et al. [25], the setback viscosity (SB) indicates an increase in the viscosity of the starch paste due to the re-polymerization of the starch molecules, especially between the amylose molecules, during the cooling process. As can be seen in Table 2, the amylose content of PS (25.50%) was higher than that of NS (23.57%) and CPS (23.77%). In general, amylose content and SB are positively correlated. The SB of PS was 16.67 mPa.s, which was much greater than that of NS (2.00 mPa.s) and CPS (3.33 mPa.s). Increased SB is indicative of a high tendency for retrogradation. These findings imply that NS and CPS have better cold paste stability as compared to PS. The lower retrograde tendency of CPS could be due to the introduction of cross-linked bonds, which reduces the rearrangement of starch molecules into ordered structures.

#### 3.1.4. Thermal Properties

The thermal characteristic parameters of starch are summarized in Table 3. As can be seen from Table 3, as compared to the conclusion temperature (Tc) and △H of NS, these parameters decreased significantly for PS, while the onset temperature (To) and peak temperature (Tp) were only slightly decreased, and the difference was not significant. The ∆H of NS (12.02 J/g) was significantly higher than that of PS (11.21 J/g), indicating that the intermolecular force of starch was weakened by enzymatic modification, and starch could be gelatinized with less heat [26]. This energy-saving feature may be beneficial for the industrial application of PS. In addition, the △H of the crystallization disappearance reaction caused by heating of starch solution is related to the structure of starch crystal [27]. The stable double helix structure can improve the thermal stability of starch, as discussed further based on FT-IR results. However, there was no significant difference in the thermodynamic parameters before and after starch cross-linking, indicating that sodium phytate as a cross-linking agent would not significantly change the thermodynamic properties of PS.

#### 3.1.5. Properties of Starch Gel

The textural parameters of starch gel are shown in Table 4. For starches from different sources, starch with more amylose and longer branches generally has higher hardness, springiness, glueyness, and chewiness [28]. In this study, the gel texture of porous starch was significantly changed compared with the *C. esculentus* starch. The hardness, springiness, glueyness, and chewiness of porous starch were significantly reduced. Among them, the decrease of hardness may be due to the damage of the rigid and ordered structure of the starch particles in the PS processing process by the complex enzyme, which leads to the break of the starch molecular chain, thus affecting the arrangement behavior of starch molecules in the forming process of gel, resulting in the decrease of hardness. The springiness of starch gel is related to water holding capacity. It has been confirmed in Section 3.1.2 that PS starch paste has poor water holding capacity and is easily separated into water layers, so has low springiness. The decrease of glueyness and chewiness can be attributed to the break of branch chain molecules of the starch after enzymatic treatment, forming multiple molecular chain structures with smaller molecular weight, and weakening of the association degree between molecular chains and the three-dimensional network structure formed by starch paste. In addition, the strength of starch gel can also be attributed to the double helix structure of the starch molecule; the denser the network of helices, the stronger the starch gel. According to the data in Table 5, it can be inferred that the experimental results in this paper are consistent with those reported by others [29]. No apparent differences were recorded in the parameters of starch gel texture before and after PS cross-linking, indicating that the modification of PS with sodium phytate as a cross-linking agent did not change the properties of PS gels.

### 3.2. Structural Characterization

#### 3.2.1. Changes in Basic Physical Properties

**Figure 2:**
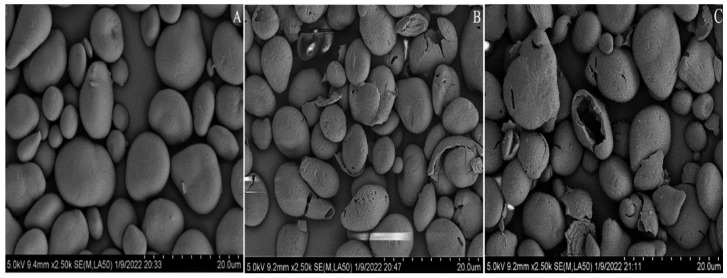
Scanning electron micrographs of native *Cyperus esculentus* starch (**A**), *Cyperus esculentus* porous starch (**B**), and *Cyperus esculentus* cross-linked porous starch (**C**).

The morphologies of *C. esculentus* starch and its modified starches visualized under SEM are displayed in Figure 2. The images show that *C. esculentus* starch was dominated by spherical starch granules with uneven sizes and relatively smooth surfaces (Figure 2A). Figure 2B shows that the enzyme treatment destroyed the original structure of starch, resulting in a significant change in the surface of the granules; the surface became rough and irregular pores appeared, which is different from the observations of Zhang et al. [30]. This discrepancy could mainly be due to the different sources of starch used and the differences in the effects produced, even when the same enzyme is used [23]. Figure 2C shows that cross-linking modification of enzyme-modified starch preserved its porous structure without causing damage.

#### 3.2.2. Crystalline Properties

**Figure 3:**
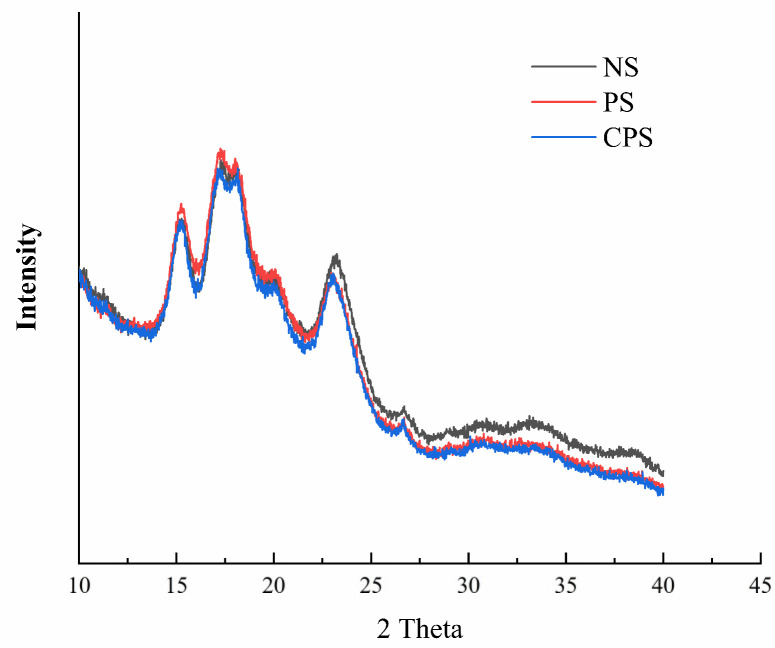
X-ray diffraction patterns. NS: native *Cyperus esculentus* starch; PS: *Cyperus esculentus* porous starch; CPS: *Cyperus esculentus* cross-linked porous starch.

Starch granules are complex polycrystalline systems, and according to the X-ray diffraction patterns, starch can be classified into A-, B-, C-, and V-types [31]. The X-ray diffraction patterns of *C. esculentus* starch and its modified form are exhibited in Figure 3. It can be seen from the figure that the two modification methods did not change the crystalline pattern of the starch, and the three starches exhibited a typical A-type crystallization pattern, with strong diffraction peaks at 2θ of 15°, 17°, 18°, and 23°, and two weak diffraction peaks at 20° and 26°. The relative crystallinity of starch is shown in Table 5. The relative crystallinity of NS was 42.36%, and that of PS and CPS was 38.22% and 34.31%, respectively. The crystallinity of starch decreased after enzymatic modification, which was due to the joint action of α-amylase and amyloglucosidase from *A. niger* on starch that broke down the starch chain and led to molecular recombination. However, a further decrease in the crystallinity of CPS relative to PS indicated that the reaction process with sodium phytate as a PS cross-linker was chemically modified, which was consistent with the results previously reported by Sun et al. [19].

#### 3.2.3. The FT-IR Spectra

**Figure 4:**
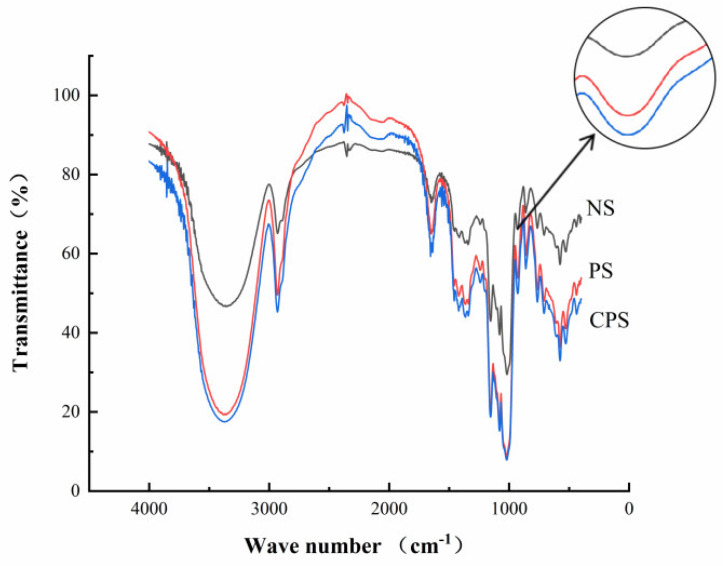
FTIR spectra. NS: native *Cyperus esculentus* starch; PS: *Cyperus esculentus* porous starch; CPS: *Cyperus esculentus* cross−linked porous starch.

The Fourier infrared spectra of *C. esculentus* starch and its modified form are shown in Figure 4. The positions of the characteristic absorption peaks of starch after enzymatic modification and cross-linking were similar to NS, indicating that there was no significant difference in the functional groups of the three starches, and that enzyme modification and cross-linking did not change the basic structure of starch [28]. The absorption peak of about 3400 cm^−1^ is attributed to the stretching vibration of the hydroxyl group, where the intensity is positively correlated with the number of intermolecular hydrogen bonds of starch. As shown in Figure 4, the strength of the absorption peak at this wave number was noticeably stronger for PS and CPS relative to NS, which was mainly because starch produces short amylose chains during enzymatic reactions, which react with free hydroxyl groups, as well as intermolecular and intramolecular hydroxyl groups, resulting in the formation of more hydrogen bonds [32].

The absorption peak near 930 cm^−1^ is one of the characteristic indexes of starch hydrophilicity. The order of strength of the absorption peak in this region was as follows: NS < PS < CPS, which indicated that both enzymatic modification and cross-linking increased the hydrophilicity of starch [33,34]. However, the characteristic absorption peaks of the C-O-P bind in phosphate esters did not appear in the CPS, probably due to the low amounts of sodium phytate introduced.

The infrared spectrum of starch typically has three absorption peaks corresponding to wave numbers of 995, 1022, and 1047 cm^−1^. The ratio, R_1047/1022_, of the absorption peak intensities at 1047 cm^−1^ and 1022 cm^−1^ reflects the orderliness of starch structure in the short-range; the larger the ratio, the higher the crystallinity. The ratio, R_995/1022_, of the absorption peak intensities at 995 cm^−1^ and 1022 cm^−1^ reflects the internal changes of the starch double helix structure. Generally, the larger the ratio, the more double-helical structures are present [35]. The results are shown in Table 5. The R_1047/1022_ and R_995/1022_ of the PS were significantly lower than that of NS, which indicated that the enzymatic modification destroyed the orderliness of starch structure in the short-range and reduced the crystallinity, which was consistent with the results of X-ray diffraction analysis and also the reason for the lower △H of PS than NS. However, the R_1047/1022_ of CPS was higher than that of PS, while R_995/1022_ was smaller than that of the PS, because not all double-helical structures can form crystals [36].

#### 3.2.4. X-ray Photoelectron Spectroscopy

**Figure 5:**
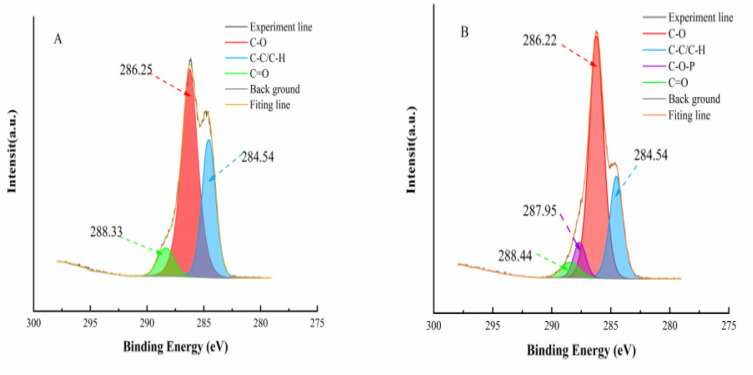
X-ray photoelectron spectroscopy of *Cyperus esculentus* porous starch (**A**) and *Cyperus esculentus* cross-linked porous starch (**B**).

The elemental composition at the surface of the starch is usually characterized by XPS. The binding energies and chemical states of C1s in PS and CPS are shown in Figure 5A.B, respectively. From Figure 5A, it can be seen that C1s in PS produced three different peaks at 284.54, 286.25, and 288.33 eV, corresponding to C-C/C-H, C-O, and C=O, respectively. However, as compared with PS, C1s in CPS (Figure 5B) produced peaks at 284.54, 286.22, 287.95, and 288.44 eV, where the new peak at 287.95 eV corresponded to the C-O-P, indicating that sodium phytate was introduced into the cross-linked porous starch and bound to it to form C-O-P bonds. This is similar to the way sodium trimetaphosphate binds to starch, as previously demonstrated by Li et al. [12].

#### 3.2.5. Specific Surface Area and Pore Size Analysis

**Figure 6:**
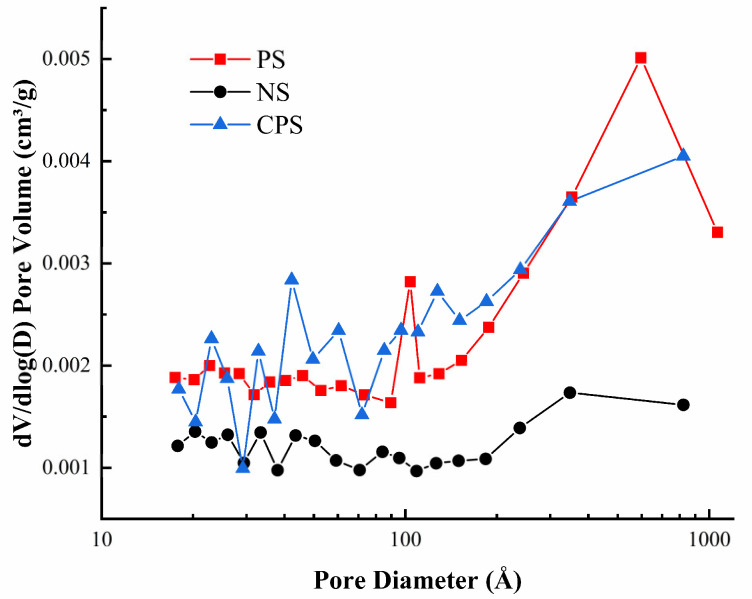
Pore size distribution curves for starch. NS: native *Cyperus esculentus* starch; PS: *Cyperus esculentus* porous starch; CPS: *Cyperus esculentus* cross-linked porous starch.

The specific surface area and pore size of starch samples were determined by the nitrogen adsorption and desorption methods. The pore size distribution of the three starches is shown in Figure 6. As can be seen from Figure 6, compared with NS, the number of pores in PS increased significantly, indicating that the enzyme treatment generated more pores on the surface of porous starch, which was consistent with the results observed by SEM [37]. Theoretically, the changing trend of increasing the number of pores will increase the specific surface area of starch (Table 5), which will lead to higher adsorption capacity, which was also verified in the results of the oil absorption capacity measurement. The pore size distribution of PS changed significantly after the cross-linking modification. The number of pores larger than 400 Å decreased relatively, while the number of pores near 100 Å increased to a certain extent. This change was attributed to the occurrence of the cross-linking reaction on the surface of the starch granules or between the internal macropores, leading to the formation of more uniform and compact pores. As can be seen from Table 5, the specific surface area of CPS is significantly increased as compared with PS, indicating that appropriate reduction of pore size contributes to the increase of specific surface area [38].

## 4. Conclusions

PS can be obtained by modifying *C. esculentus* starch with α-amylase and amyloglucosidase from *A. niger*, and then cross-linked with sodium phytate to obtain CPS. These two modification methods can significantly change many properties of *C. esculentus* starch and have great significance for expanding the application radius of *C. esculentus* starch. Finally, the study of these structural and property changes can provide a reference for the development of the starch industry.

## Figures and Tables

**Table 1:** Physical properties.

Starch	Oil Absorption (g/g)	Emulsification Capacity (mL/g)	Transmittance (%)
NS	0.5419 ± 0.0091 ^c^	4.2 ± 0.6 ^b^	10.8 ± 0.08 ^c^
PS	1.3606 ± 0.0259 ^a^	22.6 ± 1.1 ^a^	13.16 ± 0.30 ^b^
CPS	1.2744 ± 0.0158 ^b^	5.4 ± 0.6 ^b^	16.25 ± 0.81 ^a^

Different superscript letters in the same column imply significant differences at (*p* ≤ 0.05). NS: native *Cyperus esculentus* starch; PS: *Cyperus esculentus* porous starch; CPS: *Cyperus esculentus* cross-linked porous starch.

**Table 2:** Pasting properties and amylose content.

Starch	Amylose Content (%)	PV (mPa.s)	TV (mPa.s)	BD (mPa.s)	FV (mPa.s)	SB (mPa.s)
NS	23.57 ± 0.04 ^c^	235.50 ± 4.95 ^a^	231.00 ± 2.83 ^a^	4.50 ± 2.12 ^a^	233.00 ± 2.83 ^a^	2.00 ± 0.00 ^b^
PS	25.50 ± 0.03 ^a^	69.68 ± 6.51 ^c^	72.00 ± 6.56 ^c^	−2.33 ± 0.58 ^b^	88.60 ± 5.03 ^c^	16.67 ± 2.08 ^a^
CPS	23.77 ± 0.06 ^b^	121.00 ± 4.36 ^b^	113.33 ± 5.77 ^b^	7.67 ± 1.53 ^a^	116.67 ± 5.69 ^b^	3.33 ± 1.53 ^b^

Different superscript letters in the same column imply significant differences at (*p* ≤ 0.05). PV: peak viscosity; TV: trough viscosity; BD: breakdown; FV: final viscosity; SB: set back; NS: native *Cyperus esculentus* starch; PS: *Cyperus esculentus* porous starch; CPS: *Cyperus esculentus* cross-linked porous starch.

**Table 3:** Thermodynamic parameters.

Starch	To (°C)	Tp (°C)	Tc (°C)	△H (J/g)
NS	66.18 ± 0.33 ^a^	71.53 ± 0.28 ^a^	78.15 ± 0.17 ^a^	12.02 ± 0.24 ^a^
PS	65.44 ± 0.17 ^ab^	71.42 ± 0.08 ^a^	76.71 ± 0.11 ^b^	11.21 ± 0.25 ^b^
CPS	65.04 ± 0.24 ^b^	71.17 ± 0.08 ^a^	77.14 ± 0.21 ^b^	10.74 ± 0.24 ^b^

Different superscript letters in the same column imply significant differences at (*p* ≤ 0.05). To: onset temperature; Tp: peak temperature; Tc: conclusion temperature; ΔH: enthalpy change; NS: native *Cyperus esculentus* starch; PS: *Cyperus esculentus* porous starch; CPS: *Cyperus esculentus* cross-linked porous starch.

**Table 4:** A summary of gel properties.

Starch	Hardness (N)	Springiness (mm)	Glueyness (N)	Chewiness (mJ)
NS	0.835 ± 0.057 ^a^	2.718 ± 0.059 ^a^	0.651 ± 0.084 ^a^	1.774 ± 0.265 ^a^
PS	0.603 ± 0.080 ^b^	2.369 ± 0.023 ^b^	0.452 ± 0.048 ^b^	1.071 ± 0.106 ^b^
CPS	0.497 ± 0.047 ^b^	2.333 ± 0.100 ^b^	0.389 ± 0.019 ^b^	0.906 ± 0.011 ^b^

Different superscript letters in the same column imply significant differences at (*p* ≤ 0.05). NS: native *Cyperus esculentus* starch; PS: *Cyperus esculentus* porous starch; CPS: *Cyperus esculentus* cross-linked porous starch.

**Table 5:** Crystallization, surface area, and infrared (IR) ratios of starches.

Starch	Crystallinity (%)	R_1047/1022_	R_995/1022_	Surface Area (m^2^/g)
NS	42.36 ± 0.66 ^a^	0.8712 ± 0.0004 ^a^	0.928 ± 0.0070 ^a^	0.9984 ± 0.02 ^c^
PS	38.22 ± 0.64 ^b^	0.8653 ± 0.0006 ^b^	0.8198 ± 0.0063 ^b^	1.7303 ± 0.116 ^b^
CPS	34.31 ± 0.42 ^c^	0.8648 ± 0.0013 ^b^	0.8396 ± 0.0074 ^b^	2.7734 ± 0.08 5^a^

Different superscript letters in the same column imply significant differences at (*p* ≤ 0.05). To: onset temperature; Tp: peak temperature; Tc: conclusion temperature; ΔH: enthalpy change; NS: native *Cyperus esculentus* starch; PS: *Cyperus esculentus* porous starch; CPS: *Cyperus esculentus* cross-linked porous starch.

## Data Availability

The date are available from the corresponding author.
